# Supplementary material for: Oxygen vacancy-driven orbital multichannel Kondo effect in Dirac nodal line metals IrO2 and RuO2
Source: Nat Commun. 2020 Sep 21;11:4749. doi: 10.1038/s41467-020-18407-7 (PMC7506538; doi:10.1038/s41467-020-18407-7)
Supplement: Supplementary file 1 — Supplementary Information [file 41467_2020_18407_MOESM1_ESM.pdf]

SUPPLEMENTARY INFORMATION

Oxygen vacancy-driven orbital multichannel Kondo effect in Dirac nodal line metals IrO_2 and RuO_2

Yeh et al.

Supplementary Note 1 Thermal annealing effect on the number density of oxygen vacancies n_{v_O} in $M\mathrm{O}_2$ rutiles

The number density of oxygen vacancies n_{v_O} in a given sample can be tuned by thermal annealing. The annealing conditions for the $M\mathrm{O}_2$ NWs studied in this work are listed below:

- IrO_2 NW A was annealed at 300°C in vacuum for 1 h.
- IrO_2 NW B1 was annealed at 450°C in vacuum for 10 min.
- IrO_2 NW B2 was oxygenated in air for $\sim$5 months after the first measurement.
- IrO_2 NW 3 was oxygenated in air.
- RuO_2 NW A was annealed at 450°C in argon for 5 min.
- RuO_2 NWs B–E were as-grown.
- RuO_2 NW 4 was oxygenated in air.

A control experiment of RuO_2 films. In addition to NWs, we have recently grown a series of 150-nm-thick RuO_2 films and carried out a control experiment of the annealing effect under various atmospheric conditions to extract the n_{v_O} values[1]. The n_{v_O} values in the samples have been inferred from both x-ray photoelectron spectroscopy and $1/f$ noise studies. These two independent methods give consistent results. Our $1/f$ noise measurements demonstrate the relation $\gamma \propto n_{v_O}$. Here γ is the Hooge parameter which characterizes the magnitude of the voltage noise power spectrum density $S_V(f) = \gamma V^2/N_c f$, where V is the bias voltage, f is the frequency, and N_c is the total number of charge carriers in the sample. Supplementary Fig. 1 is reproduced from figure 6(a) of Ref. [1], which shows the variation of γ with T for four RuO_2 films that underwent different annealing conditions (at 500°C), as indicated. We see that annealing for 25 min in O_2 greatly reduces the γ value (blue symbols), whereas an additional 5 min annealing in Ar significantly increases the γ value (pink symbols). Thus, annealing in O_2 reduces the n_{v_O} value in the sample. In contrast, annealing in Ar increases the n_{v_O} value in the sample.

Supplementary Note 2 Extraction of the anomalous contribution to the resistivity

Our measurements of $\rho(T)$ indicate that above $\sim$a few (tens) degrees Kelvin, depending on the NWs, the variation of ρ with T in both IrO_2 and RuO_2 NWs obeys the Boltzmann transport equation, as previously established[2]. Thus, the residual resistivity ρ_{B0} (listed in Table 1) and the Debye temperature, θ_D, of each NW can be extracted by the standard method.

Supplementary Fig. 2(a) shows $\rho(T)$ data for three IrO_2 NWs. The solid curves are Boltzmann transport predictions, with the fitted values θ_D = 275, 260 and 260 K for NWs A, B1 and B2, respectively. For clarity, the data for NWs B1 and B2 are offset by 30 and 20 μΩ cm, respectively.

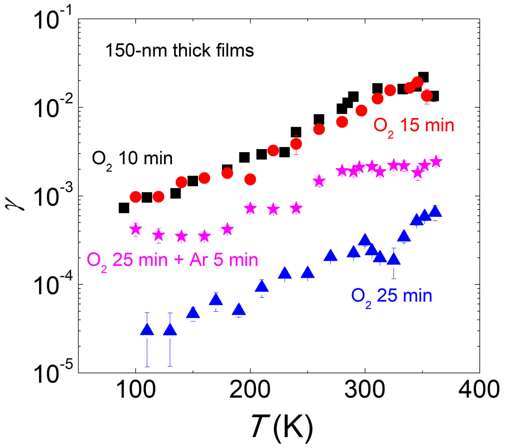

Supplementary Figure 1: **Hooge parameter versus temperature for RuO_2 films.** Description in the text.

The inset of Supplementary Fig. 2(a), plotted with a $\log T$ scale, demonstrates that the anomalous low-T (Kondo) resistivity increases with decreasing T. The data are offset by 34.7 μΩ cm (NW B1) and 33.6 μΩ cm (NW B2) for clarity.

For RuO_2 NWs, an additional term due to the coupling of electrons with optical-mode phonons needs to be included to fully describe $\rho(T)$ curves[2]. The fitted values are θ_D = 375 K and θ_E = 790 K for all NWs, where $k_B\theta_E/\hbar$ is the optical-phonon frequency. Supplementary Fig. 2(b) shows the $\rho(T)$ curves for NWs F (diameter $d = 21$ nm) and G ($d = 90$ nm), which were aged in air. The solid curves are the Boltzmann transport predictions. In these two NWs, the low-T anomalies are barely seen. Note that in a number of RuO_2 NWs we have observed pronounced temporal resistance fluctuations, associated with an increase in the time-averaged resistivity with decreasing T at low T, as previously reported[3]. Thus, the averaged resistivity $\langle\rho(T)\rangle = \frac{1}{\triangle t}\int_t^{t+\triangle t} dt'\rho(T,t')$ is our main focus, i.e., we have averaged the measured resistivities to smooth out any time-dependent fluctuations before extracting the low-T anomalies. The solid curves in the main panel of Supplementary Fig. 2(c) show the Boltzmann transport predictions for RuO_2 NWs B and C. The symbols are the measured $\langle\rho(T)\rangle$. The inset shows $\rho(T)$ on a lin-log scale to demonstrate the logarithmic behavior of the Kondo effect for T at and above T_K. (We also would like to remark that in IrO_2 NWs and in a good number of other metallic NWs, such as ITO NWs[4] and heavily indium-doped ZnO NWs[5], we have not found any noticeable temporal resistance fluctuations at low T.)

Supplementary Fig. 3 shows that the relation between our extracted ρ_{B0} and the concentration of orbital Kondo scatterers n_{V_O}, with the exception of NW B, indicates an approximately linear relation for RuO_2 NWs (cf. Supplementary Note 6 for the extraction of n_{V_O} and Table 1 for the extracted values).

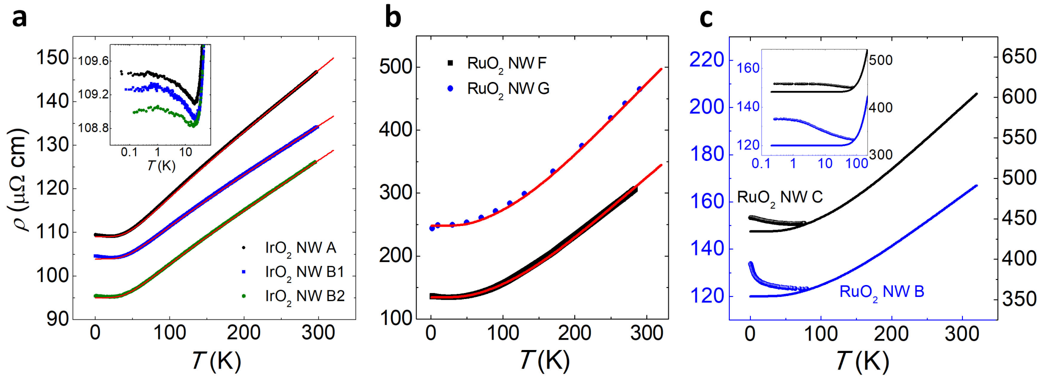

Supplementary Figure 2: **Resistivity versus temperature for IrO_2 and RuO_2 NWs.** Description in the text.

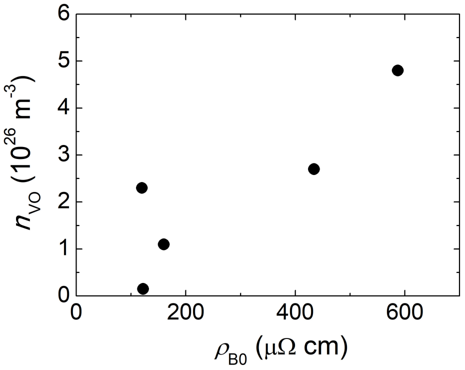

Supplementary Figure 3: **Number density of oxygen vacancies (i.e., orbital Kondo scatterers) versus residual resistivity for RuO_2 NWs.** Description in the text.

We note that, due to the presence of high levels of point defects in the MO_2 NWs studied in this work, our measured resistance ratios are small, i.e., $R(300\,\mathrm{K})/R(10\,\mathrm{K}) < 2$, in all samples, implying that the contribution of the electron-phonon scattering to the measured $\rho(T)$ below several tens degrees Kelvin is minute. This specific material property turns out to be a great advantage in the separation of the anomalous resistivity from ρ_{B0}. Quantitatively, the electron-phonon scattering contributes less than a few percent to our measured anomalous resistivity in every NW.

Supplementary Note 3 Electron-electron interaction and weak-localization effects

Here we expand on the arguments briefly presented in the main text indicating the negligible contribution of EEI effect and weak localization (WL) corrections to the low-T transport properties. In weakly disordered metals, the EEI effect generically dominates over the WL effect in causing characteristic resistivity increases at low T, which depends sensitively on the effective di-

mensionality of the metal[6]. For isotropic 3D systems like the MO_2 rutiles, this EEI correction, $\triangle\rho(T) = \rho(T) - \rho(T_0)$, possesses a $\sqrt{T}$ dependence and is given by[7]

$$\frac{\triangle\rho(T)}{\rho(T_0)} = -\frac{0.915e^2}{4\pi^2\hbar}\left(\frac{4}{3} - \frac{3}{2}\tilde{F}\right)\rho(T_0)\sqrt{\frac{k_\mathrm{B}}{\hbar D}}\left(\sqrt{T} - \sqrt{T_0}\right) , \tag{1}$$

where T_0 is a reference temperature, e is the electronic charge, $2\pi\hbar$ is the Planck constant, $\tilde{F}$ is the screening factor which parameterizes the degree of screening of the EEI effect, and D is the diffusion constant. Empirically, it has been established that, typically, $\tilde{F} \lesssim 0.1$ in weakly disordered metals and alloys[8–10]. It thus appears as if the EEI correction may offer an alternative explanation for the observed $\sqrt{T}$ transport anomaly found in IrO_2 NWs. Indeed, the explanation of a $\sqrt{T}$ resistivity anomaly at low T in terms of a nonmagnetic 2CK effect driven by a dynamical Jahn-Teller effect[11] has recently been challenged in favor of an explanation based on the Altshuler-Aronov correction[12–14]. (The samples studied in Ref. [11] were the *layered* compound $\mathrm{ZrAs}_{1.58}\mathrm{Se}_{0.39}$.) It is thus important to irrefutably connect the behavior observed in IrO_2 NWs to the orbital 2CK effect. We therefore stress that the observed resistivity rise at low T cannot be due to the EEI effect and the even smaller WL correction. These two effects would only cause a resistivity correction which would be approximately one order of magnitude smaller than the low-T anomalies observed in our measurements on IrO_2 and RuO_2 NWs[3]. For example, for the IrO_2 NW B1 with $\rho(10\,\mathrm{K})$ = 74 μΩ cm, $D \simeq$ 6.2 cm^2/s, and by taking $\tilde{F} = 0$, Supplementary Eq. (1) predicts a largest possible resistance increase of $\triangle\rho/\rho \simeq 2.8 \times 10^{-4}$ as T is cooled from 20 to 1 K. In reality, we have observed a much larger resistance increase of 5.1×10^{-3} (Fig. 2). The characteristic thermal diffusion length in the EEI effect is calculated to be $L_\mathrm{T} = \sqrt{D\hbar/k_\mathrm{B}T} = 69/\sqrt{T}$ nm (where T is in K), justifying that the NW is 3D with regard to the EEI effect.

The anomalous resistivities we observe in Figs. 2 and 3 are intrinsic properties of IrO_2 and RuO_2 rutiles, independent of sample geometry. However, in practice, the resistance of a bulk single crystal (e.g., of a volume $\sim$0.1×0.1×0.5 mm^3) will be too small for the relative resistance change due to the orbital Kondo effect to be detected unless a large current is applied, which is deemed to cause electron heating. On the other hand, the orbital Kondo resistance in a patterned IrO_2 or RuO_2 film (e.g., of a volume $\sim$0.1×1×10 μm^3) shall be readily detectable by using a small current to avoid electron overheating. Experiments on several types of MO_2 films are in progress.

Ruling out surface defect scattering scenario. In this work, the low-T resistivity anomaly measured in a given NW is a bulk property of the NW. It originates from the coupling of conduction electrons with those $\mathrm{V_O}$'s distributing across the radius of the NW. If the dynamic defect scattering were dominantly taking place near the surface of a NW, one would expect quasi-two-dimensional (quasi-2D), instead of 3D EEI effect to play a role. The resistance increase due to the quasi-2D EEI effect is given by[5,10]

$$\frac{\triangle R_\square(T)}{R_\square(T_0)} = -\frac{e^2}{2\pi^2\hbar}\left(1 - \frac{4}{3}\tilde{F}\right)R_\square(T_0)\,\ln\left(\frac{T}{T_0}\right) , \tag{2}$$

where $R_\square$ is the sheet resistance. For concreteness, assume the thickness of an active surface

defect layer, if any exists, to be of the order of the electron mean free path $\ell \sim 3$ nm ($\ll L_{\rm T}$). For a residual resistivity $\rho_{\rm B0} \sim 100$ μΩ cm, $R_\square = \rho_{\rm B0}/\ell \sim 300$ Ω. Then, taking $\tilde{F} = 0$–0.1, Supplementary Eq. (2) predicts a resistance increase $\triangle R_\square(T)/R_\square(T_0) \sim 1\%$ as T is cooled from 20 to 1 K. However, in practice, this hypothetical surface layer must be shunted by the much larger current conduction through the bulk of the NW which has a diameter $d \sim 100$ nm. Thus, this estimate should be corrected by a factor $\sim \ell/(d/2) \sim 1/20$, leading to a reduced relative sheet resistance change of $1\% \times (1/20) \sim 0.05\%$. This value is one order of magnitude smaller than what is observed in Fig. 2. Furthermore, this quasi-2D effect predicts a $\ln T$ dependence, distinct from the measured $\sqrt{T}$ characteristic. The quasi-2D WL effect will also cause a (smaller) $\ln T$ dependence which should be suppressed by a magnetic field of $\sim$a few T (Refs. [5,10]). These features are definitely not seen in Fig. 2. Thus, our measured low-T anomalies cannot be ascribed to a surface defect scattering scenario.

Other scenarios compatible with power-law in temperature behavior. A characteristic feature of Kondo physics in metals is the increasing scattering rate as T is lowered in contrast to, e.g., phonon scattering. Processes like dynamical Coulomb blockade and Luttinger liquid behavior are also associated with power-law behavior in T. These can be safely ruled out as underlying cause in our IrO_2 and RuO_2 NWs as their diameters are much larger than the electron mean free paths in these systems which renders electron transport 3D. Moreover, dynamical Coulomb blockade requires contacts with small tunneling conductances to observe the resulting power-law behavior in T and bias voltage across the junction. The resulting power-law exponent is given by the resistance in units of the quantum of resistance $h/(2e^2)$ (Ref. [15]). Changes in the diameter of a quasi-1D conductor affect the number of transmission channels coupled to the contact and thus the dynamical Coulomb blockade exponent in contrast to the universal exponent we observe. The 3D nature of our NWs also rules out impurities in Luttinger liquids as a possible cause for the observed power-law behavior. In addition, the T dependence of ρ in this case would have a complicated and impurity concentration dependent structure[16] in contrast to our observations.

Classical magnetoresistance. For a (3D) normal metal placed in a magnetic field, the classical magnetoresistance due to the Lorentz force is given by $\Delta R(B)/R(0) = (\mu B)^2 = (B/n_e e \rho_{\rm B0})^2$, where μ is the mobility. In our NWs with charge carrier concentration $n_{\rm e} \sim 1 \times 10^{28}$ m^{-3} and $\rho_{\rm B0} \sim 100$ μΩ cm, $\Delta R(B)/R(0) \sim 4 \times 10^{-7}$ (3×10^{-5}) in $B = 1$ (9) T, which can be completely ignored. For comparison, in IrO_2 and RuO_2 bulk single crystals[2] with small $\rho_{\rm B0} \lesssim 1$ μΩ cm, the classical magnetoresistance is pronounced.

Supplementary Note 4 Atomic arrangement around an oxygen vacancy in MO$_2$ rutiles

Here we provide detailed information for the lattice parameters around the oxygen vacancy $V_{\rm O1}$ depicted in Fig. 1. The distances between neighboring ions and the relevant angles surrounding this representative vacancy are indicated in Supplementary Figs. 4(a)–(c), with their respective values listed in Table 1. (a) The three $M1$, $M2$ and $M3$ ions surrounding $V_{\rm O1}$ form a (planar)

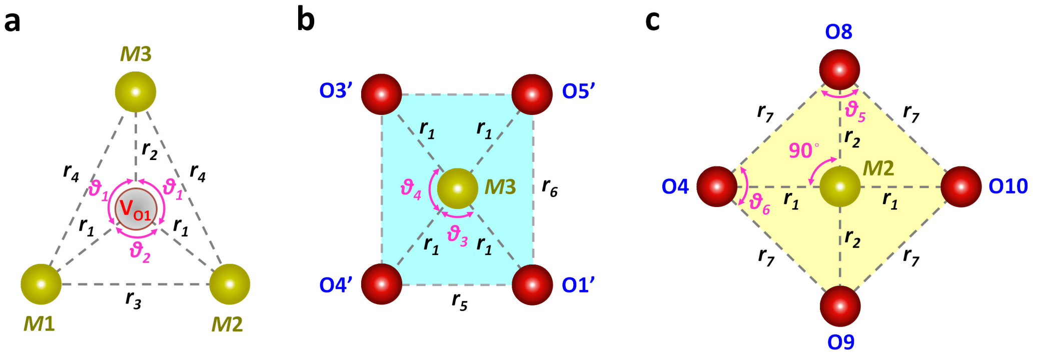

Supplementary Figure 4: **Vicinity of an oxygen vacancy.** (a) Transition metal ions $M1$, $M2$, and $M3$ in the immediate vicinity of an oxygen vacancy $V_{\mathrm{O}1}$. (b) Oxygen positions around the $M3$ site forms a rectangle. (c) The $M2$ site experiences an almost perfect C_4 symmetry despite the vicinity to $V_{\mathrm{O}1}$. Numerical values for the lattice parameters of IrO_2 and RuO_2 are provided in Table 1.

isosceles triangle. (b) The four oxygen ions, labeled O4′, O1′, O5′ and O3′, surrounding the $M3$ ion form a (planar) rectangle. (c) The four oxygen ions, labeled O4, O9, O10 and O8, surrounding the $M2$ ion form an almost perfect (planar) square. That is, r_1 differs slightly from r_2, and θ_5 and θ_6 differ slightly from 90°. These values are calculated from density functional theory and given in Refs. [17,18].

MO$_2$	r_1	r_2	r_3	r_4	r_5	r_6	r_7	θ_1	θ_2	θ_3	θ_4	θ_5	θ_6
IrO_2	2.015	1.982	3.190	3.588	2.464	3.190	2.826	127.7	104.6	75.37	104.6	90.95	89.05
RuO_2	2.006	1.964	3.140	3.576	2.497	3.140	2.807	128.5	103.0	76.99	103.0	91.20	88.80

Supplementary Table 1: **Lattice parameters around the oxygen vacancy $\mathbf{V_{O1}}$.** The interionic distances r_i ($i = 1, ..., 7$) are in Å, and the angles θ_i ($i = 1, ..., 6$) are in degree.

Supplementary Note 5 Vacancy driven 2CK and 1CK effects and dynamical large-N calculations

This section expands the discussion of orbital 1CK and 2CK physics in the DNL metals RuO_2 and IrO_2 due to V_O's in the rutile structure.

Vacancy driven orbital 2CK and 1CK effects. Our starting point is that each V_O will generate two *defect electrons*. We label the M ion sites closest to the oxygen vacancy $M1$, $M2$, and $M3$, see Supplementary Fig. 4(a). In order to minimize Coulomb repulsion, the defect electrons localize at different Mi near V_O. Model studies of V_O in TiO_2 are in line with such a picture[19–21]. A small degree of delocalization of the *defect electron* is acceptable and comparable to the behavior of the standard Anderson impurity model near its Kondo limit. For the sites $M1$, $M2$ and $M3$, the symmetry is reduced due to proximity to V_O as illustrated in Fig. 1. In contrast to the *defect*

electron localizing at the $M3$ site, the electron at either the $M2$ or $M1$ site will still experience an almost perfect $C_{4\nu}$ symmetry, see Supplementary Figs. 4(b) and 4(c). (For concreteness, we focus on the $M2$ site.) The $C_{4\nu}$ group possesses a two-dimensional irreducible representation.

We refer to the corresponding basis functions as $|\Gamma_{2\mathrm{dim}}1\rangle$ and $|\Gamma_{2\mathrm{dim}}2\rangle$ which can be taken as the components of a pseudospin variable. For the $5d$ electrons of IrO_2 (and $4d$ electrons of RuO_2), the $C_{4\nu}$ at $M2$ ensures the degeneracy of d_{xz} and d_{yz}, where the symmetry axis is parallel to the $\hat{z}$ axis. In the rutile structure, the oxygen ions above and below the square centered around $M2$, i.e., along the $\hat{z}$-axis, are minimally (less than half of their ionic radius) set off from the C_4 rotation axis. A V_O thus *enhances* the symmetry around the $M2$ site, see Fig. 1. An orthogonality theorem ensures that the conduction electrons wavefunction at the $M2$, $|\Psi\rangle$, can be decomposed in terms of the local basis functions as

$$|\Psi\rangle = \sum_{\Gamma_{n'}} \sum_{j'} f_{j'}^{(\Gamma_{n'})} |\Gamma_{n'} j'\rangle \,, \tag{3}$$

where $|\Gamma_m i\rangle$ denotes the ith basis function of the mth irreducible representation. The pseudospin associated with the two-dimensional representation is locally conserved.

In IrO_2, the valence of Ir is +IV so that the Ir ion at position $M2$ results in a [Xe]$5d^6$ electron configuration. Arguments reminiscent of those used by Cox in the context of UBe_{13} yield an effective Kondo model where the role of the nonmagnetic Γ_3 is replaced by the two-dimensional irreducible representation of $C_{4\nu}$ (Ref. [22]).

$$H = \sum_{k\sigma\mu} \epsilon_k c^\dagger_{k,\mu\sigma} c_{k,\mu\sigma} + J_{\mathrm{K}} \sum_{\substack{k,k',\sigma \\ \mu,\nu=1,2}} \vec{\tau}_d \cdot \vec{\tau}_c|_{\mu,\nu} c^\dagger_{k,\mu,\sigma} c_{k',\nu,\sigma} \,, \tag{4}$$

where $\vec{\tau}_d$ and $\vec{\tau}_c$ are vectors of Pauli matrices for d and conduction electrons in orbital space, and J_{K} is a pseudospin exchange coupling constant. The conduction electron spin projection in Supplementary Eq. (4) distinguishes the two degenerate screening channels which are necessary for obtaining an overscreened Kondo effect. If the degeneracy between the two screening channels is lifted an orbital 1CK effect ensues. This situation occurs in RuO_2. RuO_2 is an antiferromagnet with a high Néel temperature[23,24]. In RuO_2 the electron configuration of the $M2$ Ru ion is [Kr]$4d^5$. Band structure calculations show that the two t_{2g} orbitals are half-filled[24]. As a result, spin excitations of the defect electron are gapped and only orbital fluctuations are possible. These results are in line with our model for IrO_2: For $M2$ sites in the vicinity of V_O's, the orbitals d_{xz} and d_{yz}, defined with respect to the $\hat{z}$-axis shown in Fig. 1**d**, are half-filled and form the ground state for the *defect electron.* As in IrO_2, V_O's in RuO_2 trigger an orbital Kondo effect but due to the lack of time-reversal symmetry, an orbital 1CK effect occurs as demonstrated by the scaling plot of Fig. 4**a**.

The approximate nature of the almost perfect $C_{4\nu}$ symmetry implies that a low-energy scale exists below which the orbital degeneracy is lifted. We identify this scale with the deviations from the 2CK $\sqrt{T}$ behavior observed in IrO_2 NWs around $T \sim 0.5$ K, see Fig. 2. This would be less of

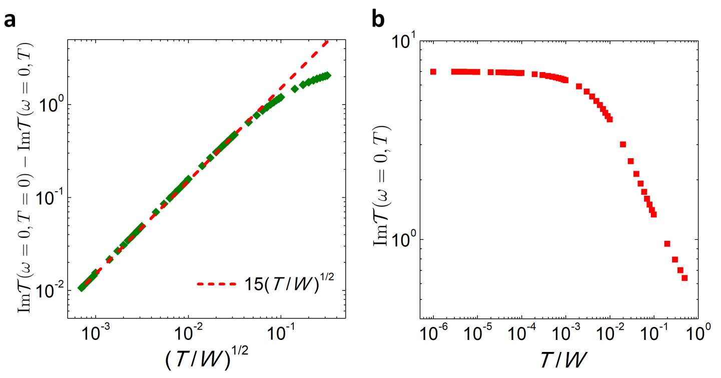

Supplementary Figure 5: **Dynamical large-N calculation results.** Imaginary part of the T-matrix (in unit of half-bandwidth W) for the multi-channel Kondo model within the dynamical large-N limit: (a) $\mathrm{Im}\mathcal{T}(\omega=0,T=0)-\mathrm{Im}\mathcal{T}(\omega=0,T)$ versus $\sqrt{T/W}$ for $\kappa=1$. This corresponds to the low-T behavior in IrO_2. (b) $\mathrm{Im}\mathcal{T}(\omega=0,T)$ for $\kappa=1/2$ which is equivalent to the one-channel case appropriate for antiferromagnetic RuO_2.

an issue for RuO_2 NWs due to the more localized nature of the $4d$ vs. the $5d$ orbitals, in line with the experimental observation.

In our arguments we focused on the defect electron localizing at site $M2$. If the second defect electron localizes at site $M1$, one may expect two-impurity Kondo physics to occur. Due to non-symmorphic rutile structure, however, the $\hat{z}$ axis with respect to which we defined d_{xz} and d_{yz} are not parallel. This fact, together with the local nature of the expansion Supplementary Eq. (3), favors local orbital Kondo screening over an orbital dimer.

Dynamical large-N calculations. The T dependence of the transport lifetime τ in our IrO_2 NWs due to orbital 2CK scatterers can be obtained, e.g., via a dynamical large-N approach. We assume that the concentration of V_O's (n_{V_O}) is small enough so that inter-vacancy effects can be ignored. The lifetime τ is related to T-matrix $\mathcal{T}(\omega,T)$ via $\tau(k)^{-1}=\frac{2}{\hbar}c_{2CK}\mathrm{Im}\langle k|\mathcal{T}(\omega,T)|k\rangle$, where c_{2CK} is the concentration of spherically symmetric dynamic scattering centers, which we identify with n_{V_O}, and $|k\rangle$ is a plane wave state. $\mathcal{T}(\omega,T)$ can be obtained from a dynamical large-N method which is known to give reliable results for multichannel Kondo, including orbital 2CK problems[22,25–27]. In this approach, the symmetry group is extended to N spin and M orbital channels, resulting in an SU(N)×SU(M) invariant Hamiltonian of the form

$$H=\sum_{k,\mu,\sigma}\varepsilon_k c^\dagger_{k,\mu,\sigma}c_{k,\mu,\sigma}+\frac{J_K}{N}\sum_{\substack{k,k'\\ \sigma,\mu,\nu}}\left(f^\dagger_\mu f_\nu-q\delta_{\mu\nu}\right)c^\dagger_{k,\mu,\sigma}c_{k'\nu,\sigma}\,, \qquad (5)$$

where μ,ν $(1,\ldots,N)$ and σ $(1,\ldots,M)$ are, respectively, the SU(M)-channel and SU(N)-spin indices and k,k' are momentum indices. The parameter κ represents the ration of spin to charge channels. For V_O's in IrO_2, $\kappa=1$ due to an equal number of spin and charge channels. If the

conduction electron spin degeneracy is lifted, as in RuO_2, $\kappa = 1/2$. Within the large-N approach, one finds

$$\mathrm{Im}\mathcal{T}(\omega, T) = -\frac{\kappa}{\pi}\int d\epsilon \Big(n_b(\epsilon, T) - n_f(\epsilon+\omega, T)\Big) A_f(\epsilon+\omega, T) A_B(\epsilon, T)\,, \tag{6}$$

where n_f (n_B) is the fermionic (bosonic) distribution function, and A_f (A_B) is the spectral function associated with the fermionic pseudoparticle representation (bosonic decoupling field)[25,27]. For $\kappa = 1$, one finds

$$\rho(T) = \rho(T=0) + A\sqrt{T} \quad \text{for} \quad T \ll T_{\mathrm{K}}\,, \tag{7}$$

with $A < 0$ as shown in Fig. 4**d**. The large-N analog of the 1CK case where $\kappa = 1/2$ is shown in Supplementary Fig. 5(b) which resembles the behavior found in RuO_2 NWs. The single-channel case is only semi-quantitatively reproduced by our dynamical large-N approach which does not capture the Fermi-liquid fixed point. We do compare the RuO_2 data to NRG calculations by T. Costi[28].

Supplementary Note 6 Extraction of the number density of oxygen vacancies $\mathrm{n_{v_O}}$

The extraction of the $\mathrm{n_{v_O}}$ values in our NWs is described below. As discussed, each V_O contributes one *defect electron* to the $M2$ (or $M1$) ion, causing the orbital Kondo effect. Because IrO_2 NWs manifest the 2CK effect, the $\rho \propto \sqrt{T}$ characteristic at low T can be compared with the dynamical large-N calculations (Sec. Supplementary Note 5), which predicts the 2CK resistivity to be

$$\rho_{\mathrm{2CK}}(T) = \frac{m}{n_e e^2}\frac{\mathrm{n_{v_O}}}{\hbar}\, 2\Lambda_a\, \mathrm{Im}\mathcal{T}(\omega=0,\, T)\,, \tag{8}$$

where $m \sim 1.6\, m_0$ is the effective electron mass (m_0 is the free electron mass)[29], $n_e \sim 1 \times 10^{28}$ $\mathrm{m^{-3}}$ is the carrier density[30], $\Lambda_a \sim 1 \times 10^{-29}$ $\mathrm{m^{-3}}$ is the atomic volume, and $\mathrm{Im}\mathcal{T}(\omega = 0, T)$ is the imaginary part of the T-matrix calculated from the dynamical large-N method. The $\mathrm{n_{v_O}}$ value of every IrO_2 NW has been extracted by fitting the measured slope of the $\rho \propto \sqrt{T}$ curve in Fig. 2 to Supplementary Eq. (8). The extracted value is in good agreement with that inferred from the conformal-field-theory calculations[31].

As the low-T resistivities of RuO_2 NWs conform to the 1CK scaling form, the ρ_{K0} value in every NW can be inferred. In this unitary limit, the Kondo resistivity is given by[32]

$$\rho_{\mathrm{K0}} = \frac{m}{n_e e^2}\frac{5\pi \mathrm{n_{v_O}}}{12\hbar g_0}\,, \tag{9}$$

where $m \sim 1.4\, m_0$ (Ref. [33]), $n_e \sim 1 \times 10^{28}$ $\mathrm{m^{-3}}$ (Ref. [34]), and the density of states per spin orientation $g_0 \sim 1 \times 10^{47}$ $\mathrm{J^{-1}\ m^{-3}}$ (Ref. [33]). The $\mathrm{n_{v_O}}$ value in every RuO_2 NW has been calculated from the extracted ρ_{K0} value, according to Supplementary Eq. (9).

Supplementary Note 7 Kondo temperature distribution

Our experimental findings are compatible with a single energy scale, termed Kondo temperature $T_{\rm K}$, that characterizes the low-T transport in each NW. This implies that the distribution of Kondo temperatures in each NW is sharply peaked around a single $T_{\rm K}$. This is demonstrated in Supplementary Fig. 6 where the measured $\langle \rho_{\rm K}(T) \rangle$ of RuO_2 NW C is plotted against NRG results for the T-matrix $\mathcal{T}(T)$ of the 1CK Kondo model, with the least-squares fitted $T_{\rm K} = 70$ K (solid curve). In addition, we plot $\mathcal{T}(T)$ with four selected $T_{\rm K}$ values (dashed curves) for comparison. Obviously, the theoretical curves with $T_{\rm K} = 35$ K and $T_{\rm K} = 105$ K already deviate significantly from the experimental data.

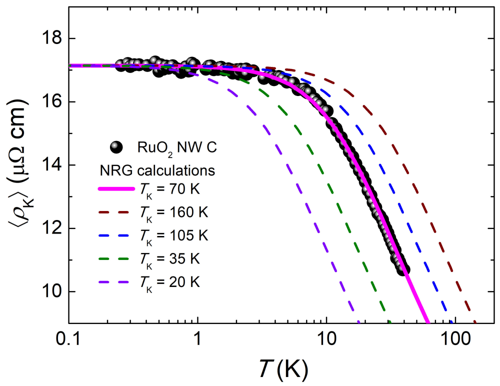

Supplementary Figure 6: **Kondo temperature extraction.** NRG calculations for the 1CK model are fitted to $\langle \rho_{\rm K}(T) \rangle$ of RuO_2 NW C with the Kondo temperature $T_{\rm K}$ as the only adjustable parameter. Solid and dashed curves are characterized by different $T_{\rm K}$ values, as indicated.

The Kondo energy scale $T_{\rm K}$ thus seems to be unaffected by the disorder generated through variations accompanying the oxygen defects. Similar observations have been reported in a variety of systems, including metal nanoconstrictions[35], dilute magnetic noble metals[36], and other Kondo systems[37,38]. This insensitivity of $T_{\rm K}$ is not entirely unexpected. The exchange coupling constant $J_{\rm K}$, Supplementary Eq. (4), reflects the wavefunction overlap between the local basis functions of the dynamic scattering center and the conduction electrons. $J_{\rm K}$ is therefore not affected by background variations due to nearby $V_{\rm O}$'s or the presence of the second *defect electron* on a nearby M ion site. This form of disorder will instead lead to local variations of the background potential. Thus, the situation resembles that considered by Chakravarty and Nayak, who have shown that for weak disorder the distribution of the local density of states is very narrow[39]. Furthermore, the thermodynamics is unaffected near the strong-coupling limit. While $T_{\rm K}$ is unaffected by the background variations caused by the weak disorder, it does change from NW to NW. These variations arise from changes in $\rho(E_{\rm F})$ as a result of the stress that exists in each NW, where $\rho(E_{\rm F})$ is the local density of states of conduction electrons coupling to the orbital degree. As the bulk and shear modulus of RuO_2 is less than those of IrO_2 (Refs. [17,18]), it appears natural that the correspond-

ing variation in $\rho(E_{\rm F})$ is bigger in RuO_2 than in IrO_2. The observed bigger variations of $T_{\rm K}$ for different RuO_2 NWs compared to those for IrO_2 NWs are consistent with this picture.

Supplementary References

1. S.-S. Yeh, K. H. Gao, T.-L. Wu, T.-K. Su, and J.-J. Lin, Phys. Rev. Appl. **10**, 034004 (2018).
2. J. J. Lin et al., J. Phys.: Condens. Matter **16**, 8035 (2004).
3. A.-S. Lien, L. Y. Wang, C. S. Chu, and J.-J. Lin, Phys. Rev. B **84**, 155432 (2011).
4. S.-P. Chiu et al., Nanotechnology **20**, 105203 (2009).
5. S.-P. Chiu, J. G. Lu, and J.-J. Lin, Nanotechnology **24**, 245203 (2013).
6. B. L. Altshuler and A. G. Aronov, *Electron-electron interaction in disordered conductors*, book section 1, North-Holland Physics Publishing, Amsterdam, The Netherlands, 1985.
7. P. A. Lee and T. V. Ramakrishnan, Rev. Mod. Phys. **57**, 287 (1985).
8. E. Akkermans and G. Montambaux, *Mesoscopic Physics of electrons and photons*, Cambridge University Press, 2006.
9. S. M. Huang, T. C. Lee, H. Akimoto, K. Kono, and J. J. Lin, Phys. Rev. Lett. **99**, 046601 (2007).
10. J. J. Lin and N. Giordano, Phys. Rev. B **35**, 545 (1987).
11. T. Cichorek et al., Phys. Rev. Lett. **117**, 106601 (2016).
12. D. Gnida, Phys. Rev. Lett. **118**, 259701 (2017).
13. T. Cichorek et al., Phys. Rev. Lett. **118**, 259702 (2017).
14. D. Gnida, Phys. Rev. B **97**, 134201 (2018).
15. G.-L. Ingold and Y. V. Nazarov, *Charge Tunneling Rates in Ultrasmall Junctions*, book section 1, Plenum Press, New York, 1992.
16. A. Furusaki and N. Nagaosa, Phys. Rev. B **47**, 4631 (1993).
17. K. Persson, Materials data on IrO_2 (sg:136) by materials project, https://doi.org/10.17188/1201432.
18. K. Persson, Materials data on RuO_2 (sg:136) by materials project, https://doi.org/10.17188/1307989.

19. B. J. Morgan and G. W. Watson, J. Phys. Chem. C **114** (2010).

20. C. Lin, D. Shin, and A. A. Demkova, J. Appl. Phys. **117**, 225703 (2015).

21. F. Lechermann, W. Heckel, O. Kristanovski, and S. Müller, Phys. Rev. B **95**, 195159 (2017).

22. D. Cox and A. Ruckenstein, Phys. Rev. Lett. **71**, 1613 (1993).

23. Z. H. Zhu et al., Phys. Rev. Lett. **122**, 017202 (2019).

24. T. Berlijn et al., Phys. Rev. Lett. **118**, 077201 (2017).

25. O. Parcollet and A. Georges, Phys. Rev. B **58**, 3794 (1998).

26. F. Zamani, T. Chowdhury, P. Ribeiro, K. Ingersent, and S. Kirchner, physica status solidi (b) **250**, 547 (2013).

27. F. Zamani, *Local quantum criticality in and out of equilibrium*, PhD thesis, TU Dresden, 2016.

28. T. A. Costi, A. C. Hewson, and V. Zlatic, J. Phys.: Condens. Matter **6**, 2519 (1994).

29. W. S. Choi et al., Phys. Rev. B **74**, 205117 (2006).

30. J. K. Kawasaki et al., Phys. Rev. Lett. **121**, 176802 (2018).

31. I. Affleck and A. W. Ludwig, Phys. Rev. B **48**, 7297 (1993).

32. K. Vladár and A. Zawadowski, Phys. Rev. B **28**, 1596 (1983).

33. K. M. Glassford and J. R. Chelikowsky, Phys. Rev. B **47**, 1732 (1993).

34. W.-C. Shin and S.-G. Yoon, J. Electrochem. Soc. (1997).

35. D. C. Ralph and R. A. Buhrman, Phys. Rev. Lett. **69**, 2118 (1992).

36. F. Mallet et al., Phys. Rev. Lett. **97**, 226804 (2006).

37. M. D. Daybell and W. A. Steyert, Phys. Rev. Lett. **18**, 398 (1967).

38. J. F. DiTusa, K. Lin, M. Park, M. S. Isaacson, and J. M. Parpia, Phys. Rev. Lett. **68**, 678 (1992).

39. S. Chakravarty and C. Nayak, Int. J. Mod. Phys. B **14**, 1421 (2000).
